# Supplementary material for: A TNFRSF14-FcɛRI-mast cell pathway contributes to development of multiple features of asthma pathology in mice
Source: Nat Commun. 2016 Dec 16;7:13696. doi: 10.1038/ncomms13696 (PMC5171877; doi:10.1038/ncomms13696)
Supplement: Supplementary Information — Supplementary Figures. [file ncomms13696-s1.pdf]

Supplementary Fig. 1

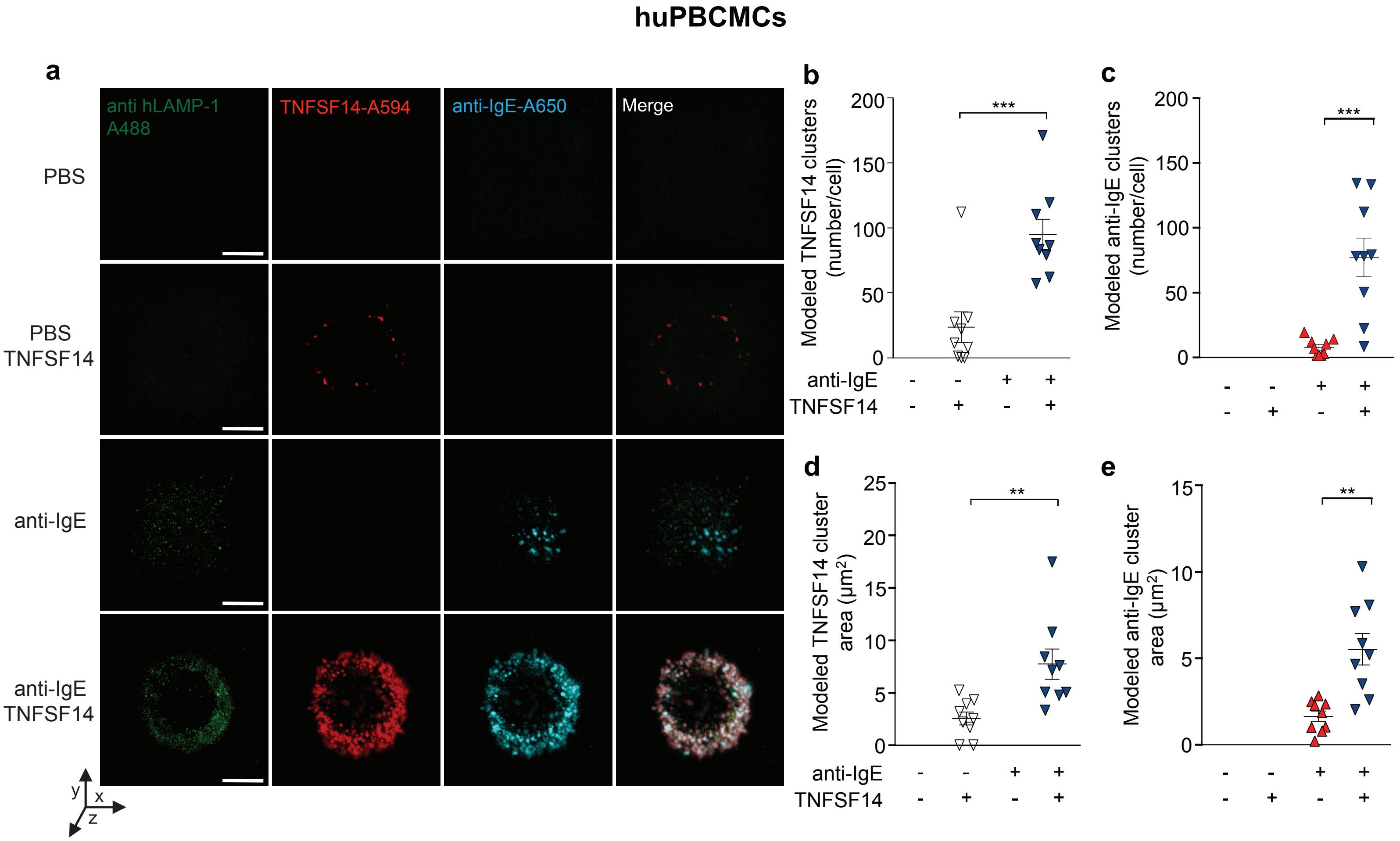

Supplementary Fig. 1. Enhanced IgE-dependent response to FcεRI crosslinking and IgE-dependent signaling upon engagement of huPBCMCs TNFRSF14 by TNFSF14. (a) Representative 3D pictures of single IgE-sensitized huPBCMCs, unstimulated or stimulated with A650-coupled anti-IgE (anti-IgE A650, cyan) in the absence or presence of human A594-coupled TNFSF14 (TNFSF14-A594, red); exocytosis of granules is detected using FITC-coupled anti-human [h]LAMP-1 [green] added before stimulation; 3D pictures were acquired 30 minutes after stimulation. Scale bar: 5 μm. (b and c) Modeled numbers of TNFSF14-A594 (b) and of anti-IgE-A650 (c) clusters per huPBCMC measured 30 minutes after stimulation. (d and e) Modeled area of individual detected clusters of TNFSF14-A594 (d) and of anti-IgE-A650 (e), measured 30 minutes after stimulation. Data shown in b-e are calculated from one representative experiment from 9 single huPBCMCs analyzed for each condition. This analysis was conducted on 3 independent experiments for all conditions shown, each of which gave similar results. In b-e, data (mean + S.E.M.) were assessed for statistical significance using a 2-tailed Student's *t* test or a Mann-Whitney test (for data with a non-normal distribution). Asterisks indicate statistical significance of differences between indicated groups. **, $p < 0.01$; ***, $p < 0.001$.

Supplementary Fig. 2

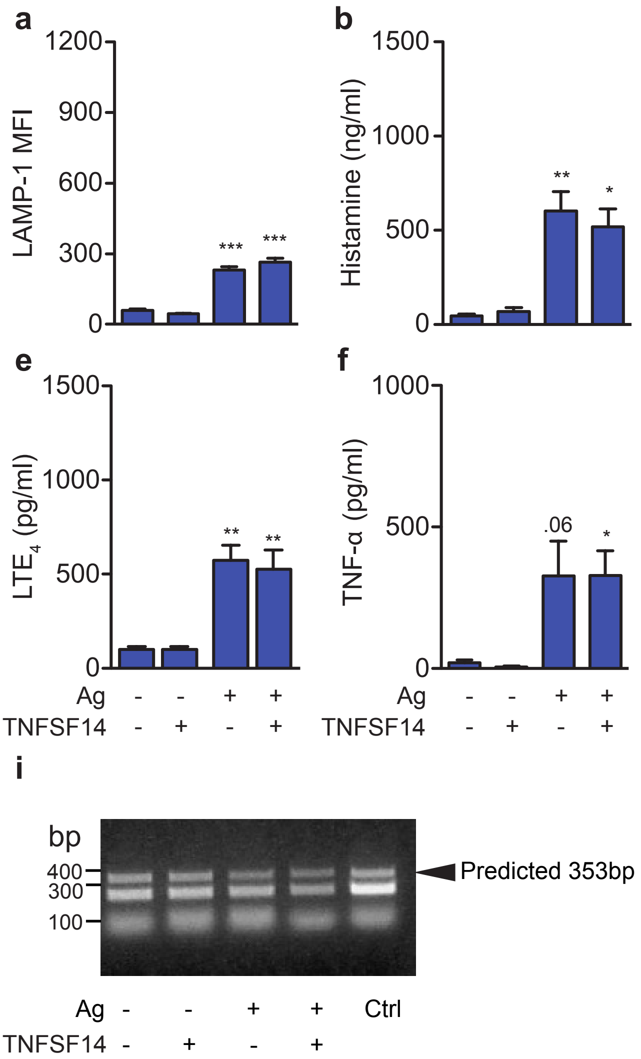

Supplementary Fig. 2. (a) LAMP-1 MFI, and (b-h) levels of histamine (b), TNF-α [early 'pre-stored', panel (c), later '*de novo* synthesized', panel (f)], LTC_4 (d), LTE_4 (e), IL-6 (g) and IL-13 (h) measured in the supernatants of IgE presensitized-*Tnfrsf14*$^{-/-}$ BMCMCs, with or without Ag stimulation in the absence or presence of TNFSF14. Results are pooled from at least 5 independent experiments, each of which gave similar results. Asterisks indicate statistical significance of differences between Ag-treated BMCMCs and the corresponding non-Ag-stimulated group. *, $p < 0.05$; **, $p < 0.01$; ***, $p < 0.001$. All data are presented as mean + or ± S.E.M and statistical significance was assessed using a Student's *t* test (2-tailed). (i) *Tnfsf14* mRNA was detectable in IgE pre-sensitized *Tnfrsf14*$^{+/+}$ BMCMCs with or without Ag stimulation in the absence or presence of TNFSF14. RNA extracted from lungs of *Tnfrsf14*$^{+/+}$ mice obtained at the end of the asthma protocol was used as a positive control (Ctrl).

Supplementary Fig. 3

PCR gel
primers: mouse TNFSF14

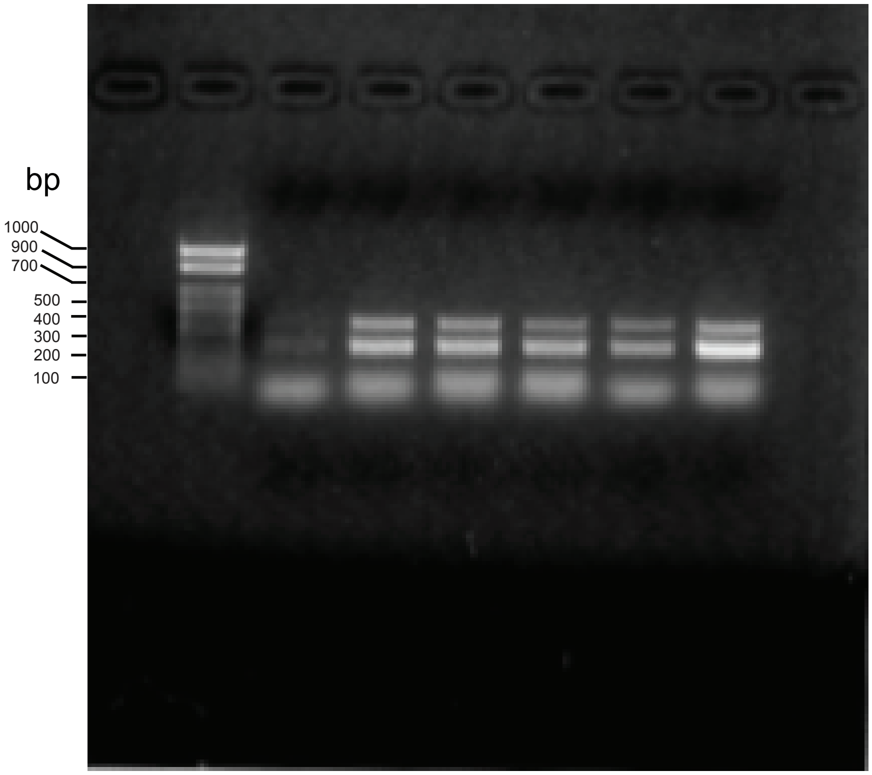

Supplementary Fig. 3. Unedited scanned photograph of the gel shown in Supplementary Fig. 2i.

Supplementary Fig. 4

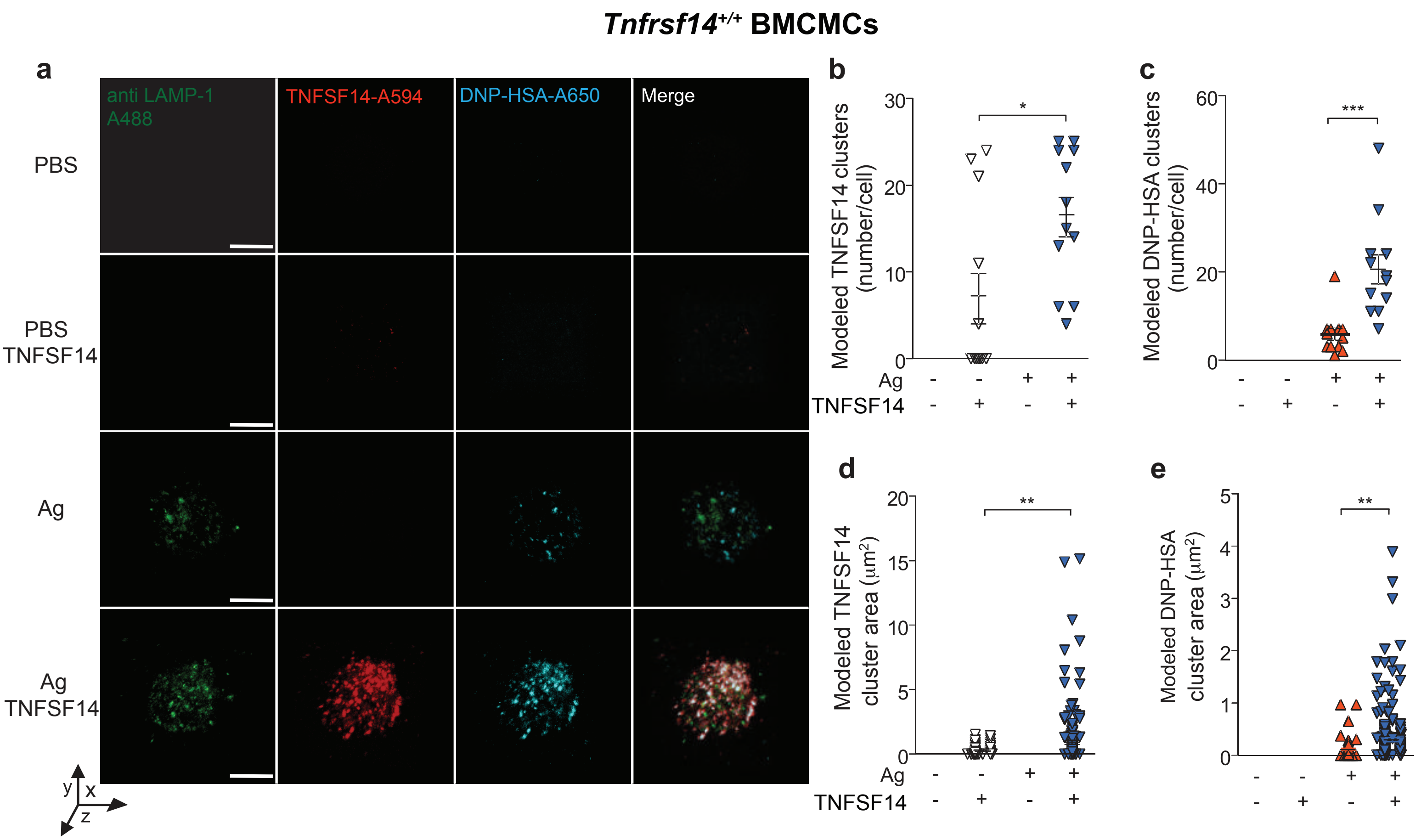

Supplementary Fig. 4. Enhanced IgE-dependent response to Ag and IgE-dependent signaling upon engagement of BMCMC TNFRSF14 by TNFSF14. (a) Representative 3D pictures of single IgE-presensitized *Tnfrsf14*$^{+/+}$ BMCMCs, unstimulated or stimulated with A650-coupled DNP-HSA (DNP-HSA-A650, cyan) in the absence or presence of mouse A594-coupled TNFSF14 (TNFSF14-A594, red); exocytosis of granules is detected using FITC-coupled anti-mouse LAMP-1 [green] added before stimulation; 3D pictures were acquired 5 minutes after stimulation. Scale bar: 5 μm. (b and c) Modeled numbers of TNFSF14-A594 (b) and of DNP-HSA-A650 (c) clusters per BMCMC measured 5 minutes after stimulation. (d and e) Modeled area of individual detected clusters of TNFSF14-A594 (d) and of DNP-HSA-A650 (e), measured 5 minutes after stimulation. Data shown in b-e are calculated from one representative experiment from at least 10 single BMCMCs analyzed for each condition. This analysis was conducted on 3 independent experiments for all conditions shown, each of which gave similar results. In b-e, data (mean + S.E.M.) were assessed for statistical significance using a 2-tailed Student's *t* test or a Mann-Whitney test (for data with a non-normal distribution). Asterisks indicate statistical significance of differences between the indicated groups. *, $p < 0.05$; **, $p < 0.01$; ***, $p < 0.001$.

Supplementary Fig. 5

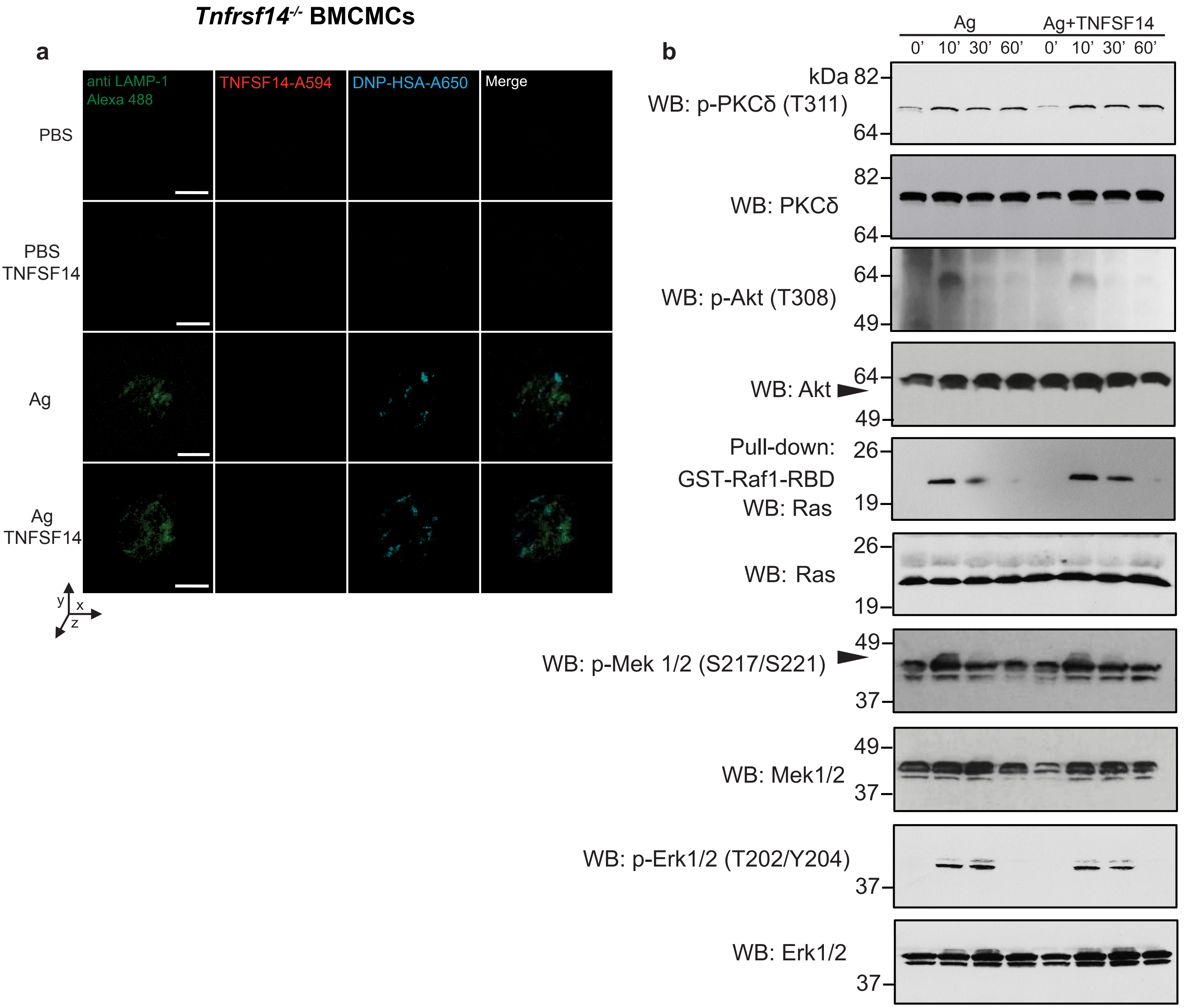

Supplementary Fig. 5. TNFSF14 does not activate *Tnfrsf14*$^{-/-}$ BMCMCs. (a) Representative 3D pictures of a single IgE-presensitized *Tnfrsf14*$^{-/-}$ BMCMC unstimulated or stimulated with Ag (DNP-HSA A650, cyan) in the absence or presence of TNFSF14-A594 (red) and exocytosis of granules (detected by the presence of LAMP-1 [green]), at a representative time point (5 min) after each type of treatment. Scale bar: 5 μm. (b) Detection of phosphorylated Akt, PKC-δ, Mek, Erk1/2 and pulled-down Ras, 0, 10, 30, 60 min after Ag stimulation or Ag/ TNFSF14 co-stimulation of IgE presensitized-*Tnfrsf14*$^{-/-}$ BMCMCs. Blots were stripped and reprobed with antibodies recognizing total levels of Akt, PKC-δ, Mek, and Erk1/2 for loading control. Loading control for Ras was collected from total BMCMC lysate before GST-pulldown. Results shown are representative of 3 independent experiments, each of which gave similar results.

Supplementary Fig. 6

Tnfrsf14$^{+/+}$ BMCMCs treated with TNFSF14 without Ag or with PMA/I

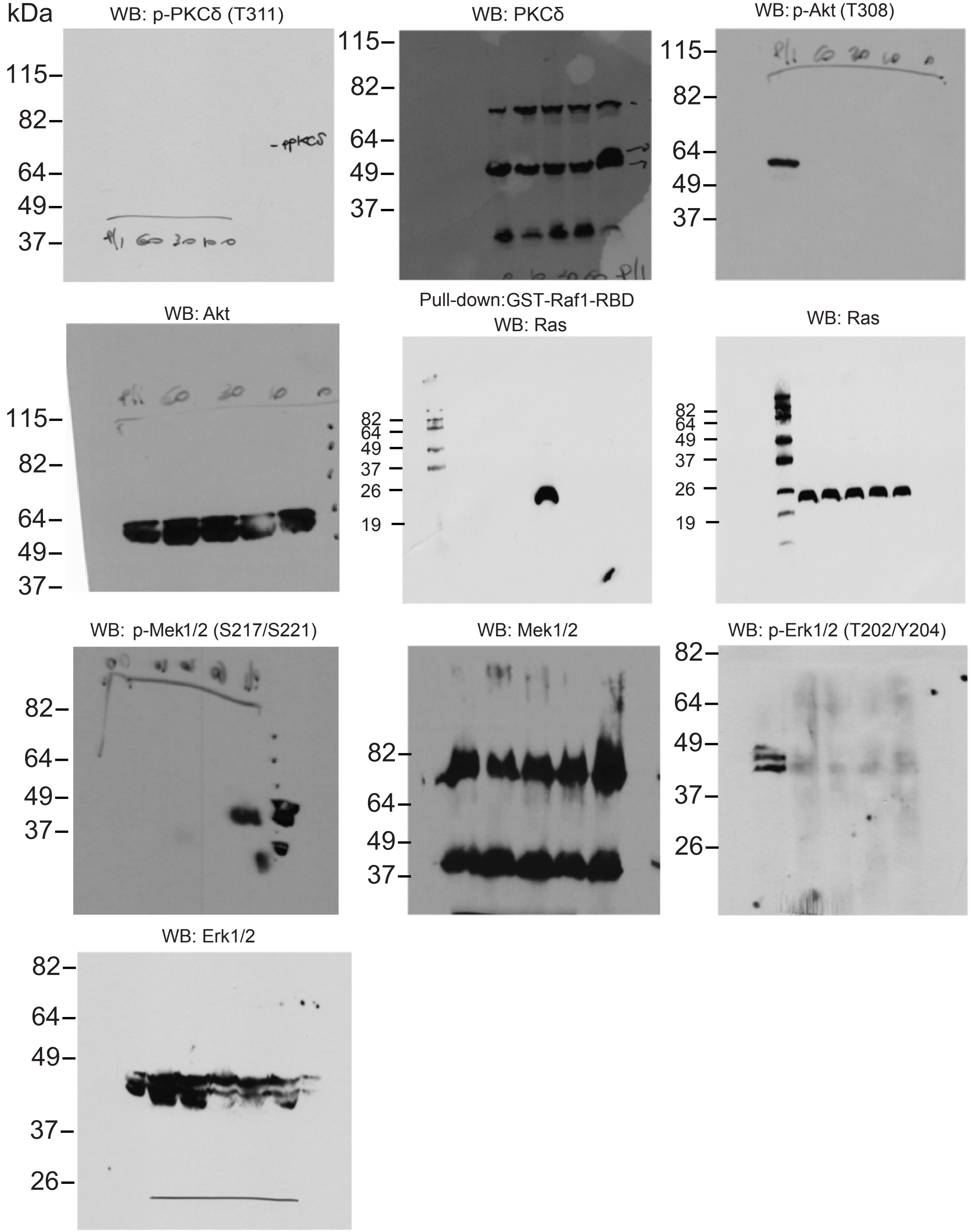

Supplementary Fig. 6. Unedited scanned photographs of the gels used to produce the Western Blot figure shown in Fig. 2a.

Supplementary Fig. 7

Tnfrsf14$^{+/+}$ BMCMCs treated with Ag with or without TNFSF14

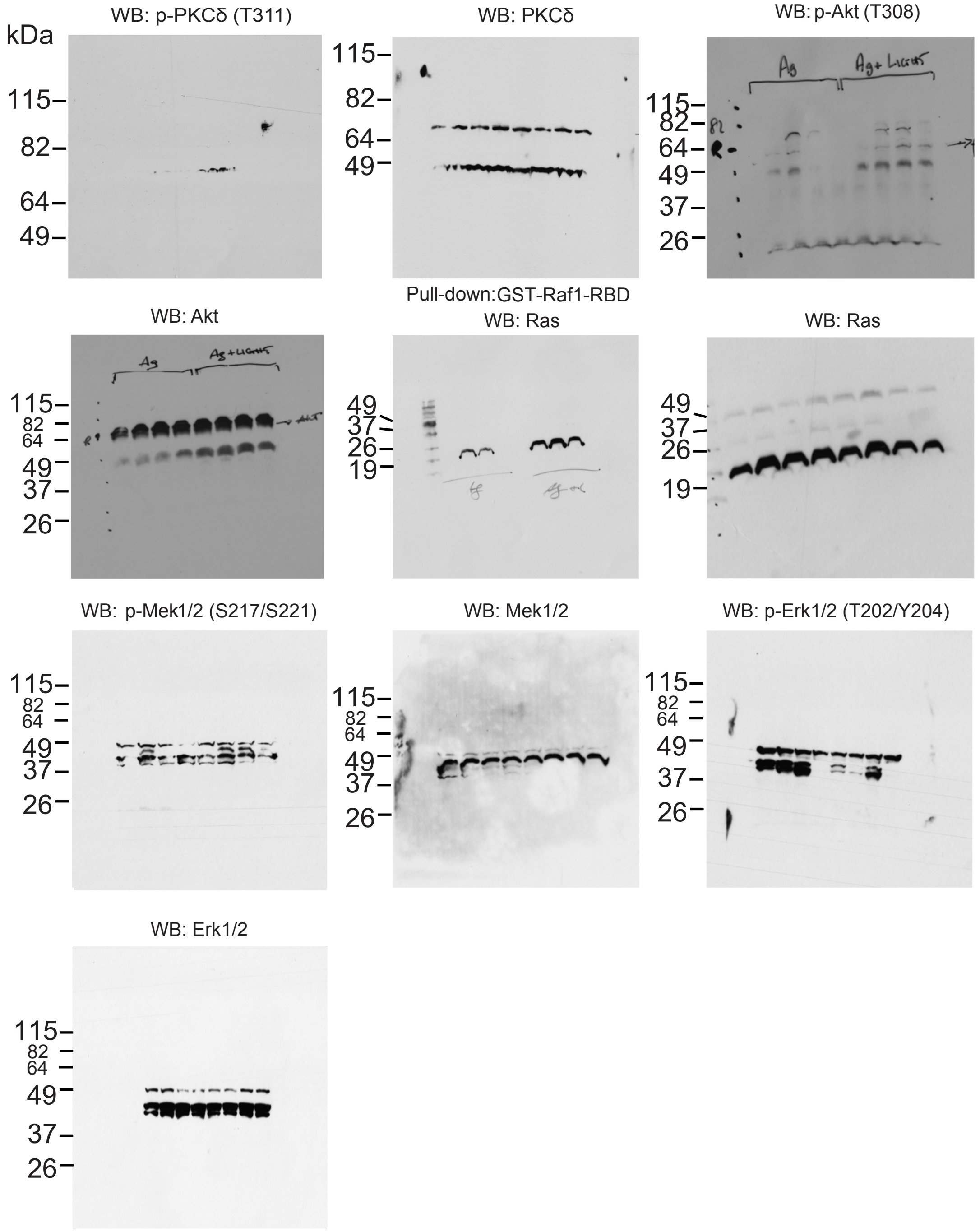

Supplementary Fig. 7. Unedited scanned photographs of the gels used to produce the Western Blot figure shown in Fig. 2b.

Supplementary Fig. 8

Tnfrsf14$^{-/-}$ BMCMCs treated with Ag with or without TNFSF14

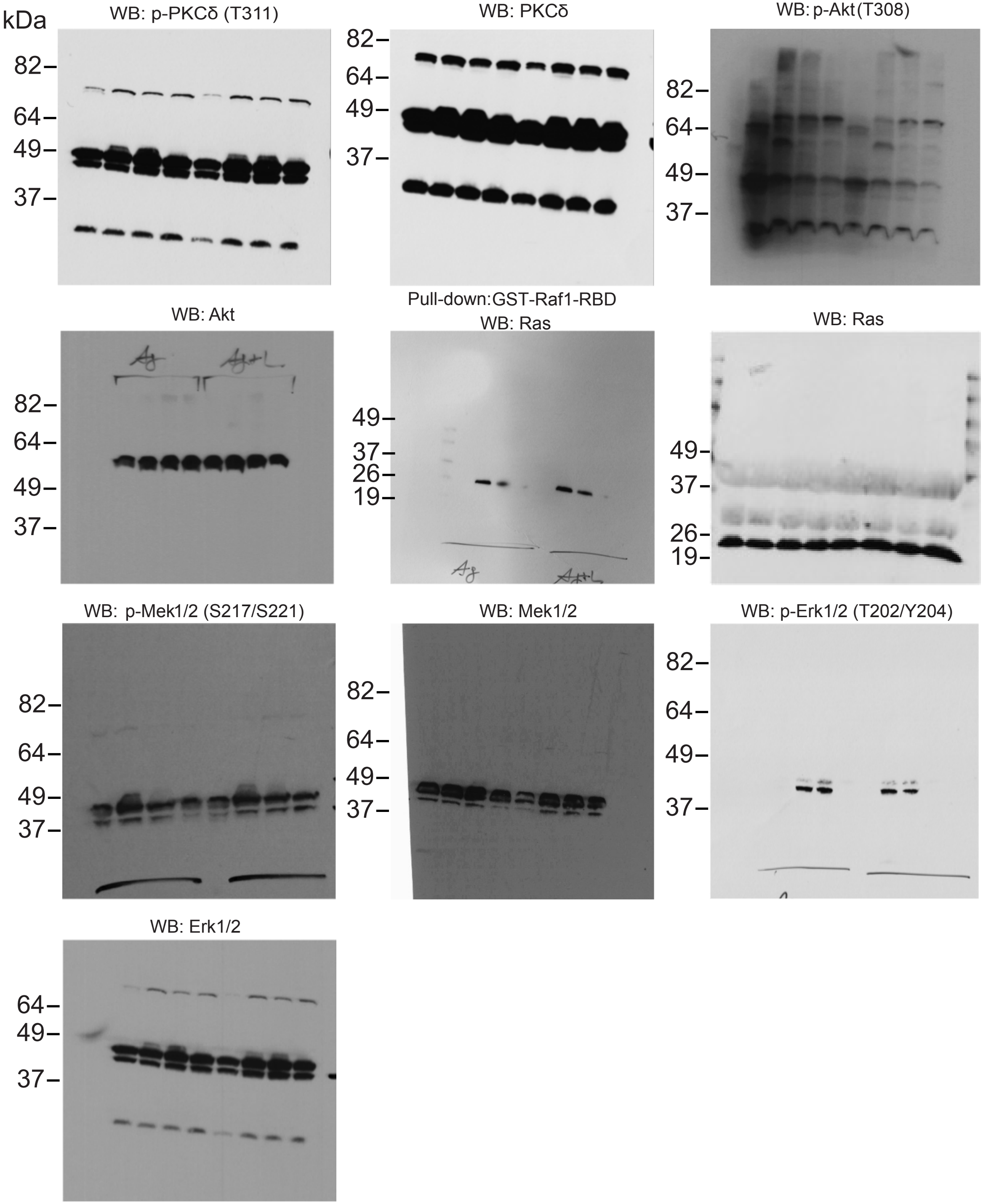

Supplementary Fig. 8. Unedited scanned photographs of the gels used to produce the Western Blot figure shown in Supplementary Fig. 5b.

Supplementary Fig. 9

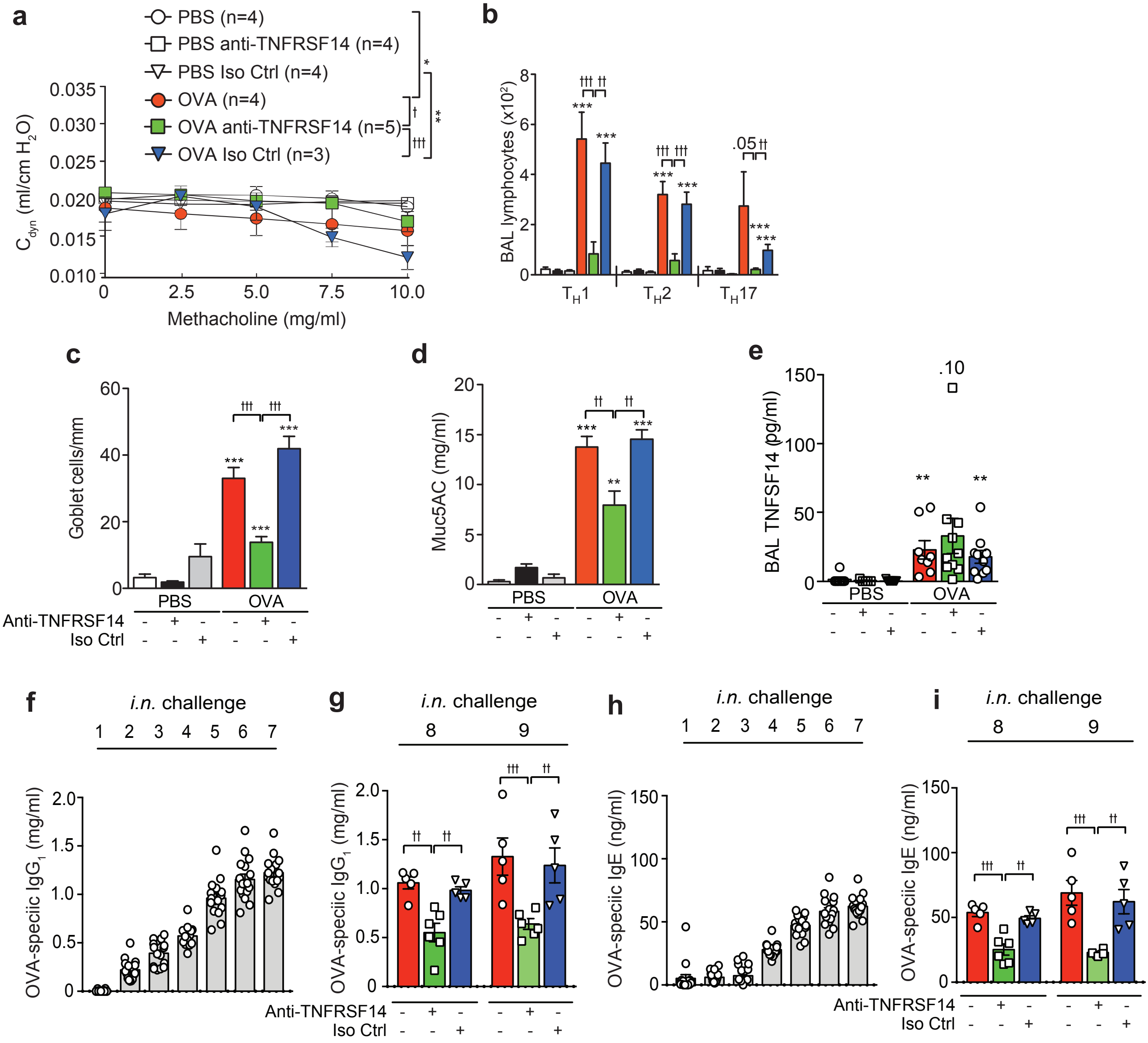

Supplementary Fig. 9. (a) Changes in C_{dyn} induced by aerosolized methacholine (Mch) 24 h after the ninth OVA or PBS challenge. (b) Numbers of T_H1, T_H2, T_H17 lymphocytes (T_H, T helper), (c) numbers of lung goblet cells and concentrations of (d) mucin (Muc) 5AC and (e) TNFSF14 in the BAL fluid, 24 h after the ninth OVA or PBS challenge in C57BL/6J mice. Data are from the same mice and treatment groups shown in Figure 3. Unless otherwise indicated, measurements were obtained from the following numbers of mice: PBS n=8, anti-TNFRSF14 PBS n=5, isotype control PBS n=9, OVA n=8, anti-TNFRSF14 OVA n=10, isotype control OVA n=10. (f-i) Blood concentrations of OVA-specific IgG_1 (f) and OVA-specific IgE (h) 1 h after the first-to-the seventh *i.n.* OVA challenge (n=16 mice in a single experiment). Blood concentrations of OVA-specific IgG_1 (g) or OVA-specific IgE (i) in mice from the same group shown in f and h, but after being divided into three groups for treatment as indicated (no Ab treatment, n=5; anti-TNFRSF14 Ab treatment, n=6; Iso Ctrl Ab, n=5) 1 h after the eighth and the ninth *i.n.* OVA challenge. Asterisks indicate statistical significance of differences between PBS-treated and the corresponding OVA-treated group; daggers indicate statistical significance of differences between indicated groups. * or †, p <0.05; ** or ††; p <0.01; *** or †††, p <0.001. All data are presented as mean + or ± S.E.M. and statistical significance was assessed using a 2-tailed Student's *t* test or a 2-way ANOVA test.

Supplementary Fig. 10

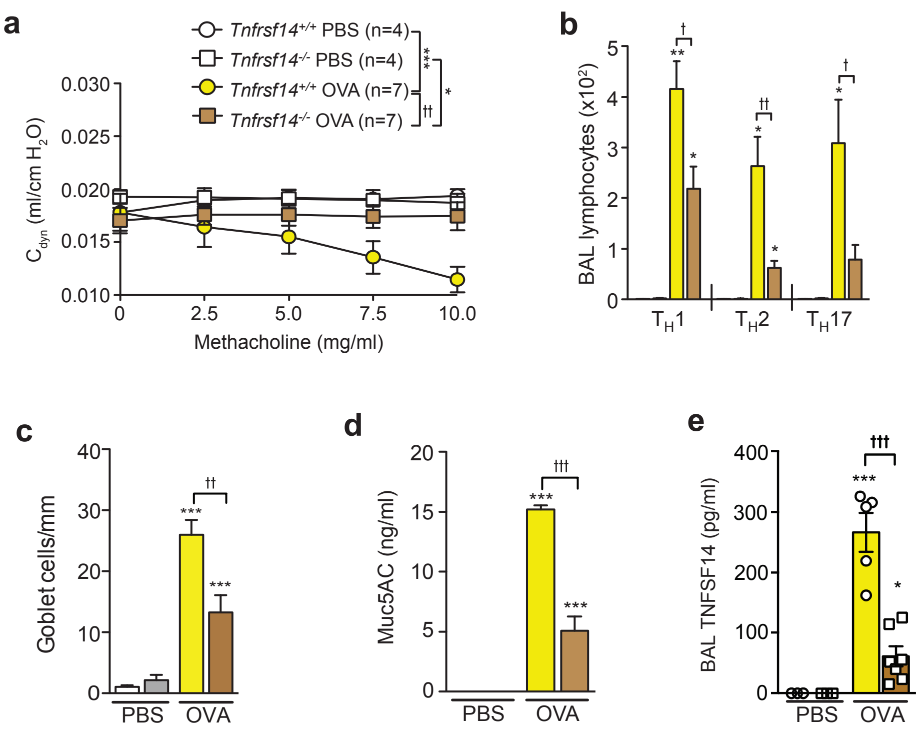

Supplementary Fig. 10. (a) Changes in C_{dyn} induced by aerosolized methacholine 24 h after the ninth OVA or PBS challenge in *Tnfrsf14*$^{-/-}$ and littermate control *Tnfrsf14*$^{+/+}$ mice. (b) numbers of T_H1, T_H2, T_H17 lymphocytes, (c) numbers of lung goblet cells and concentrations of (d) Muc5AC and (e) TNFSF14 in the BAL fluid, 24 h after the ninth OVA or PBS challenge in *Tnfrsf14*$^{+/+}$ or *Tnfrsf14*$^{-/-}$ mice. Data are from the same mice and treatment groups shown in Figure 3. Unless otherwise indicated, measurements were obtained from the following numbers of mice: *Tnfrsf14*$^{+/+}$ PBS n=3, *Tnfrsf14*$^{-/-}$ PBS n=3, *Tnfrsf14*$^{+/+}$ OVA n=5, *Tnfrsf14*$^{-/-}$ OVA n=7. In panels (c-e), asterisks indicate statistical significance between PBS-treated and corresponding OVA-treated groups and daggers indicate statistical significance between indicated groups. * or †, p <0.05; ** or ††, p <0.01; *** or †††, p <0.001. All data are presented as mean + or ± S.E.M. and statistical significance was assessed using a 2-tailed Student's *t* test or a 2-way ANOVA test.

Supplementary Fig. 11

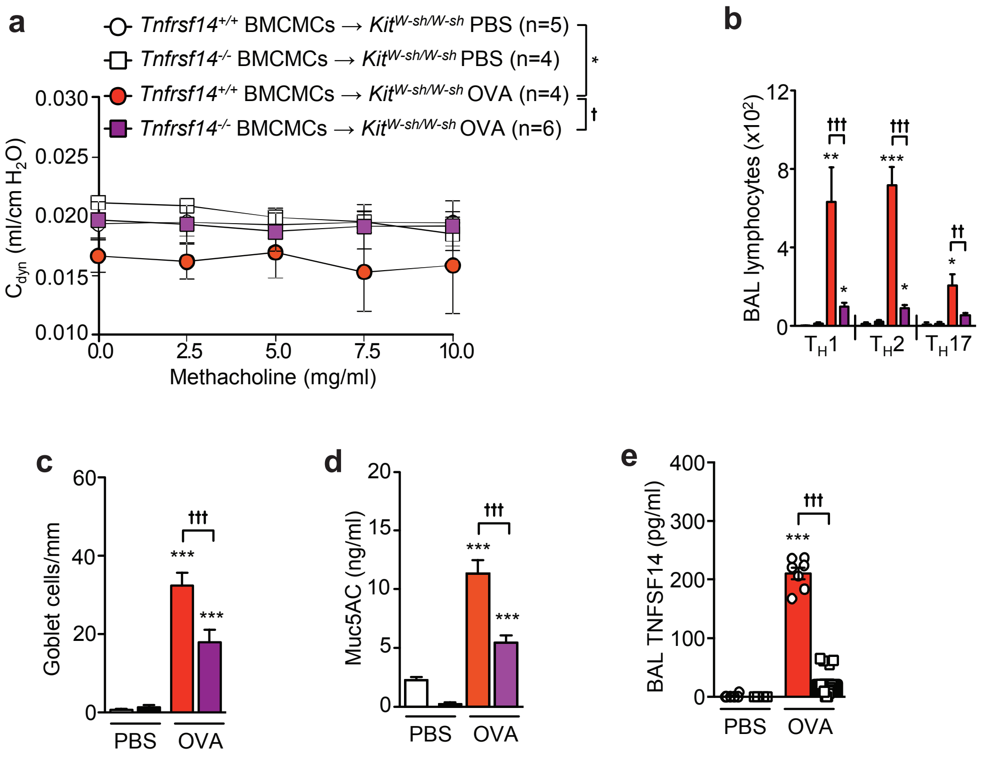

Supplementary Fig. 11. (a) Changes in C_{dyn} induced by aerosolized methacholine 24 h after the ninth OVA or PBS challenge in *Kit* mutant MC-deficient mice ($Kit^{W\text{-}sh/W\text{-}sh}$) engrafted with $Tnfrsf14^{+/+}$ or $Tnfrsf14^{-/-}$ BMCMCs ($Tnfrsf14^{+/+}$ BMCMCs→$Kit^{W\text{-}sh/W\text{-}sh}$ and $Tnfrsf14^{-/-}$ BMCMCs→$Kit^{W\text{-}sh/W\text{-}sh}$). (b) Numbers of lymphocyte subsets in BAL fluid, (c) numbers of lung goblet cells and concentrations of (d) Muc5AC and (e) TNFSF14 in BAL fluid, 24 h after the ninth OVA (or PBS) challenge. In panels (b-d), asterisks indicate statistical significance between PBS-treated and corresponding OVA-treated groups and daggers indicate statistical significance between indicated groups. * or †, $p < 0.05$; ** or ††, $p < 0.01$; *** or †††, $p < 0.001$. All data are presented as mean + or ± S.E.M. and statistical significance was assessed using a 2-tailed Student's *t* test or a 2-way ANOVA test.

Supplementary Fig. 12

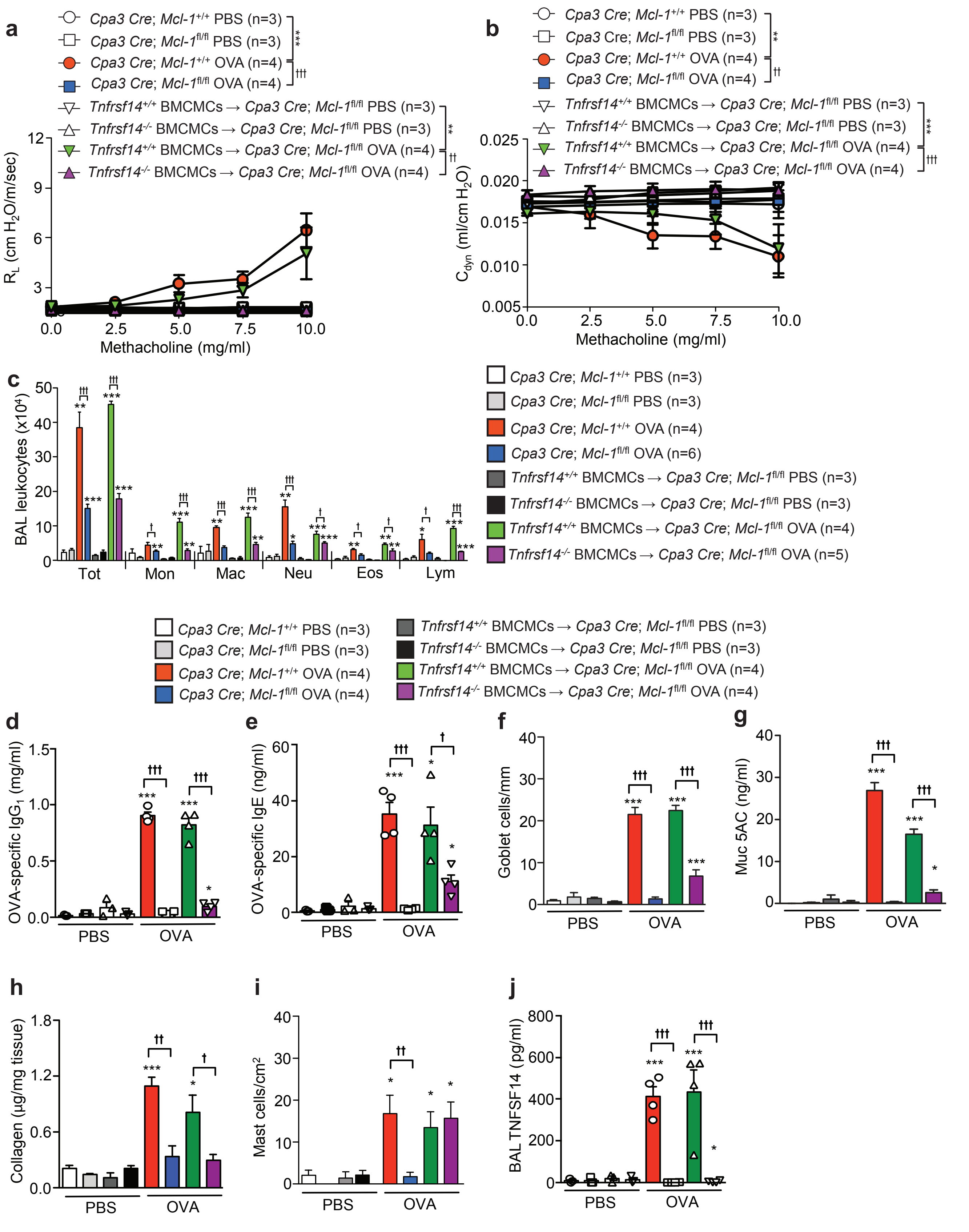

Supplementary Fig. 12. MC-deficient C57BL/6J *Cpa3 Cre;Mcl-1*$^{fl/fl}$ mice and MC-sufficient controls (*Cpa3 Cre;Mcl-1*$^{+/+}$ mice), and *Cpa3 Cre;Mcl-1*$^{fl/fl}$ mice (Lilla et al., 2011), engrafted with *Tnfrsf14*$^{+/+}$ or *Tnfrsf14*$^{-/-}$ BMCMCs (*Tnfrsf14*$^{+/+}$ BMCMCs→*Cpa3 Cre; Mcl-1*$^{fl/fl}$ or *Tnfrsf14*$^{-/-}$ BMCMCs→*Cpa3 Cre; Mcl-1*$^{fl/fl}$ mice), were subjected to the protocol of OVA-induced chronic allergic airway inflammation shown in Figure 2a. (a, b) Changes in R_L (a) and C_{dyn} (b) induced by aerosolized methacholine, (c) numbers of leukocytes in the BAL fluid, (d, e) Levels of plasma (d) OVA-specific IgG_1 and (e) OVA-specific IgE, (f) numbers of lung goblet cells, (g) levels of Muc5AC in the BAL fluid, (h) lung collagen levels, (i) numbers of lung mast cells and (j) concentrations of BAL TNFSF14 24 h after the ninth OVA or PBS challenge for the indicated groups of mice. Results are pooled from 2 independent experiments, each of which gave similar results. Asterisks indicate statistical significance between PBS-treated and corresponding OVA-treated groups and daggers indicate statistical significance between indicated groups. * or †, p <0.05; ** or ††, p <0.01; *** or †††, p <0.001. All data are presented as mean + or ± S.E.M. and statistical significance was assessed using a 2-tailed Student's *t* test or a 2-way ANOVA test.

Supplementary Fig. 13

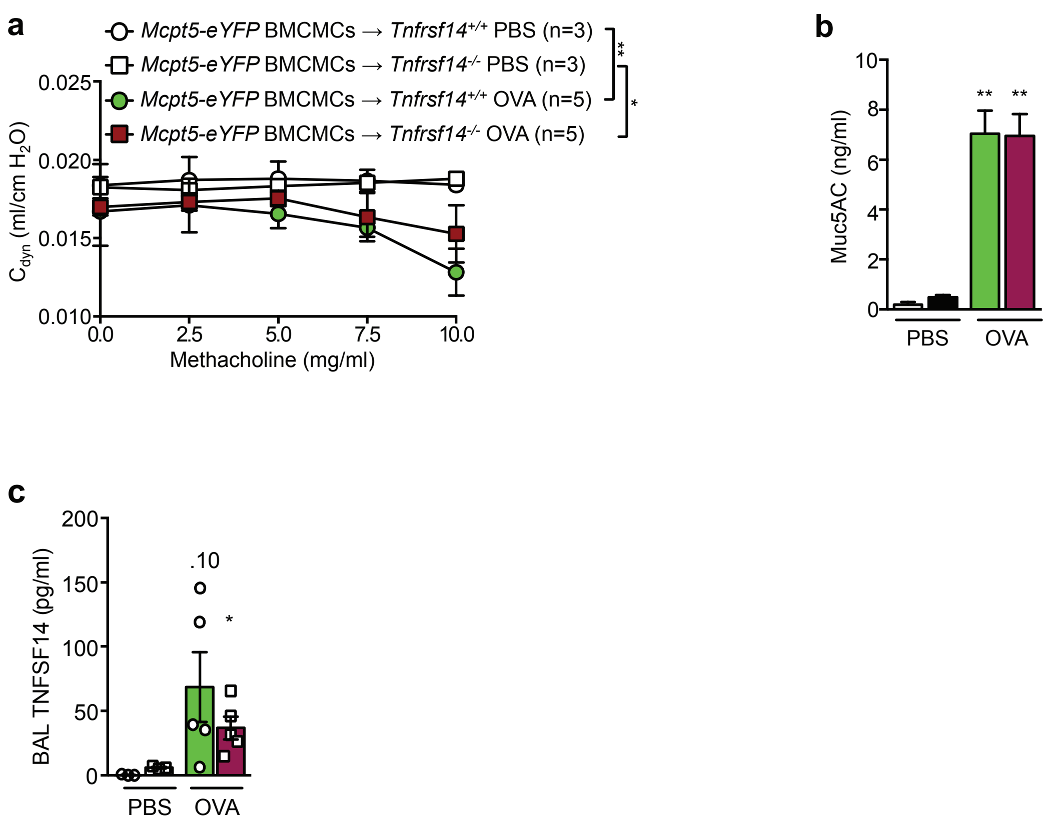

Supplementary Fig. 13. (a) Changes in C_{dyn} induced by aerosolized methacholine 24 h after the ninth OVA or PBS challenge in *Tnfrsf14*$^{+/+}$ or *Tnfrsf14*$^{-/-}$ mice engrafted with *Mcpt5-eYFP* BMCMCs (*Mcpt5-eYFP* BMCMCs→*Tnfrsf14*$^{+/+}$ and *Mcpt5-eYFP* BMCMCs→*Tnfrsf14*$^{-/-}$). (b and c) Concentrations of BAL Muc5AC (b) and TNFSF14 (c) 24 h after the ninth OVA (or PBS) challenge. Results are pooled from 2 independent experiments, each of which gave similar results. Asterisks indicate statistical significance between PBS-treated and corresponding OVA-treated groups. *, $p < 0.05$; **, $p < 0.01$. All data are presented as mean + or ± S.E.M. and statistical significance was assessed using a 2-tailed Student's *t* test or a 2-way ANOVA test.

Supplementary Fig. 14

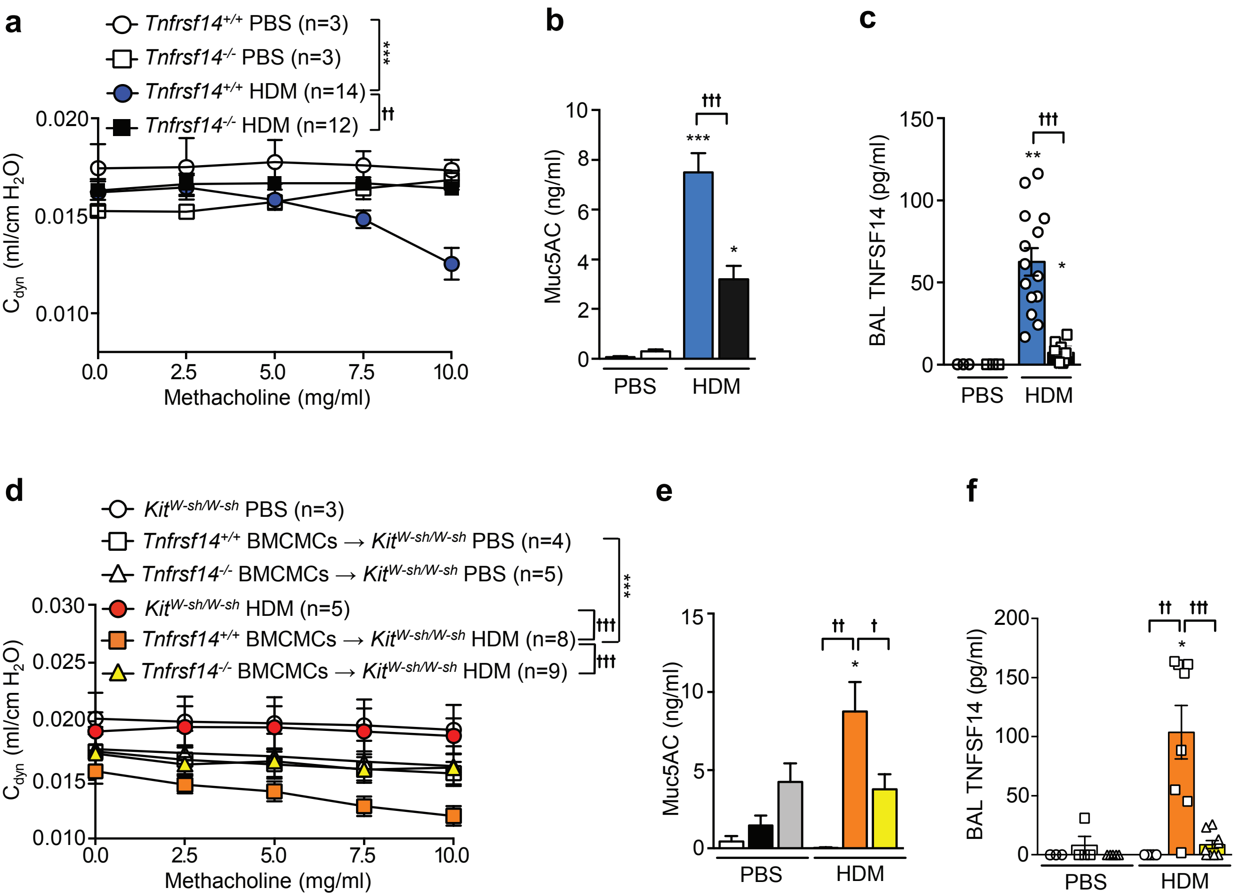

Supplementary Fig. 14. (a) Changes in C_{dyn} induced by aerosolized methacholine and concentrations of BAL (b) Muc5AC and (c) TNFSF14, 24 hours after the tenth HDM or PBS challenge in *Tnfrsf14*$^{-/-}$ and *Tnfrsf14*$^{+/+}$mice. (d) Changes in C_{dyn} induced by aerosolized methacholine and concentrations of BAL (e) Muc5AC and (f) TNFSF14, 24 h after the tenth HDM (or PBS) challenge in MC-deficient *Kit*$^{W\text{-}sh/W\text{-}sh}$ mice and in *Kit*$^{W\text{-}sh/W\text{-}sh}$ mice engrafted with *Tnfrsf14*$^{+/+}$ or *Tnfrsf14*$^{-/-}$ BMCMCs. Numbers of mice for each condition are indicated. Results are pooled from at least 2 independent experiments, each of which gave similar results. Asterisks indicate statistical significance between PBS-treated and corresponding HDM-treated groups and daggers indicate statistical significance between indicated groups. * or †, $p < 0.05$; ** or ††, $p < 0.01$; *** or †††, $p < 0.001$. All data are presented as mean + or ± S.E.M. and statistical significance was assessed using a 2-tailed Student's *t* test or a 2-way ANOVA test.
